# Aversion to a High Salt Taste is Disturbed in Patients With CKD

**DOI:** 10.1016/j.ekir.2024.02.1393

**Published:** 2024-02-16

**Authors:** Natsuko Okuno-Ozeki, Yusuke Kohama, Hiromu Taguchi, Yuka Kawate, Minato Umehara, Atsushi Minamida, Hiroko Yamauchi-Sawada, Yasuto Sunahara, Yayoi Matoba, Itaru Nakamura, Kunihiro Nakai, Tomohiro Nakata, Yuhei Kirita, Takuya Taniguchi, Keiichi Tamagaki, Takashi Hirao, Satoaki Matoba, Tetsuro Kusaba

**Affiliations:** 1 Department of Nephrology, Graduate School of Medical Science, Kyoto Prefectural University of Medicine, Kyoto, Japan; 2 Research and Development Headquarters, House Foods Group Inc. Chiba, Japan; 3 Department of Nutrition, Kyoto Katsura Hospital, Kyoto, Japan; 4 Department of Cardiovascular Medicine, Graduate School of Medical Science, Kyoto Prefectural University of Medicine, Kyoto, Japan

**Keywords:** aversion threshold, chronic kidney disease, salt taste, taste test

## Abstract

**Introduction:**

A reduced salt intake is a vital lifestyle modification in the management of hypertension. Initiatives aimed at decreasing the intake of salt are based on the preference by humans for a salt taste. Salt intake behavior appears to be affected by the balance between attraction to a low salt taste and aversion to a high salt taste. However, aversion to a high salt taste has not yet been quantitively investigated in both healthy individuals and patients with chronic kidney disease (CKD).

**Methods:**

Assessments of gustatory and aversion thresholds for salt, bitter, sour, and sweet tastes were performed using a stimulant-impregnated test strip in healthy subjects and patients with CKD.

**Results:**

In a pilot taste test of 125 healthy subjects, the number of participants with an aversive reaction increased at higher salt concentrations. The threshold for normal taste perception was arbitrarily defined as 10% NaCl, with 47.2% of healthy subjects displaying an aversive reaction. In taste tests performed by 70 patients with CKD, 10% were unable to recognize a salt taste, even at the highest concentration (20% NaCl), suggesting a significant impairment in taste perception in patients with CKD. Only 15.7% of patients with CKD exhibited a normal aversion to NaCl, whereas 78.6% showed the complete loss of aversion to salt.

**Conclusion:**

The present results confirmed the anticipated aversive response to a high salt taste in humans and demonstrated its impairment in patients with CKD, implying that patients with CKD have reduced resistance to a high salt intake.

## Graphical abstract

**Figure:**
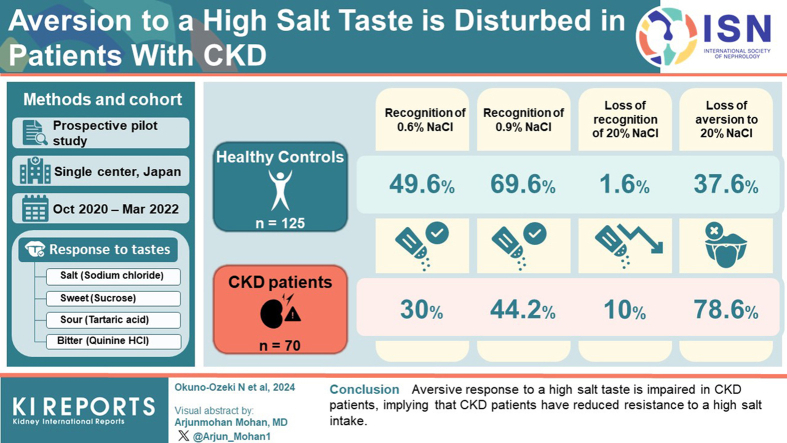

The number of patients with CKD is increasing worldwide,^1^ and hypertension is the leading global risk factor for cardiovascular diseases and CKD.^2^ Salt intake is a major factor affecting blood pressure, and many epidemiological studies have shown a strong positive correlation between salt intake and blood pressure.^3^ Therefore, a restricted salt intake is important for appropriate blood pressure control, and the World Health Organization and several guidelines recommend a daily salt intake of lower than 5 g/d^4^, ^5^, ^6^, ^7^; however, most individuals consume more salt than recommended,^8^ and are considered to be increasing their own risk of cardiovascular diseases and CKD progression.

Current approaches to reducing salt intake are based on the concept that humans are primitively attracted to a salt taste; however, this is not always the case. As evidenced by our repulsion to drinking seawater, high concentrations of salt elicit behavioral aversion. Regarding the molecular mechanisms and signaling pathways involved in the regulation of salt intake preferences in mammals, taste stimulation via the taste buds plays a crucial role.^9^, ^10^, ^11^ Two distinct mechanisms underlie cellular sensitivity to a salt taste in humans and rodents: epithelial sodium channels (ENaC) that are sensitive and insensitive to amiloride, an ENaC inhibitor.^12^ Regarding the former, the ENaC-dependent salt taste is sensitive to low salt concentrations (<100 mM) and is involved in salt taste attraction, which is attenuated in ENaC-KO mice.^12^ Alternatively, the response to a stimulation with a high concentration of salt is not attenuated by amiloride, which indicates an ENaC-independent salt taste transduction pathway.^13^^,^^14^ This pathway is considered to play a role in aversive behavior towards a high salt taste, which is elicited via the activation of bitter and sour taste receptors when loaded with high salt concentrations.^15^ Transmembrane channel 4, expressed in taste cells, was recently shown to be responsible for an ENaC-independent high salt taste preference.^16^

Based on these findings, salt intake in mammals is assumed to be affected by the appetitive-aversive balance, for example, for low salt concentrations (accelerator) and the avoidance of high salt concentrations (decelerator). Most of the taste tests previously conducted on humans analyzed salt taste recognition or preferences under specific disease conditions^17^; however, not many quantitatively examined aversive responses to a high concentration of salt.^18^ To elucidate the aversive behavior of salt, it is necessary to study not only the salt taste, but also its interactions with other tastes. In the present study, we attempted to quantitatively evaluate aversive behavior to high salt concentrations in healthy subjects by adding the concepts of the preference for and aversion to salt. Moreover, because taste disorders are common in patients with CKD,^17^^,^^19^, ^20^, ^21^, ^22^, ^23^, ^24^, ^25^, ^26^ we performed taste tests on patients with CKD.

## Methods

### Subjects

We recruited patients with CKD attending or hospitalized at the Department of Nephrology, Kyoto Prefectural University Hospital between October 1, 2020 and March 31, 2022 as participants in this cross-sectional study. Normal subjects were recruited from the general public between October 1, 2020 and March 31, 2022. Subjects who were staff or students of our institution were selected in accordance with the general recruitment and limited to other departments and were considered to be intrainterested parties.

Participation requirements for both patients with CKD and healthy subjects were aged ≥20 years and the provision of consent to participate in the study. Regarding healthy subjects, the results of an annual health check-up, which is widely performed on the general population in Japan, were checked and those with CKD, generally defined as an abnormal urinalysis and estimated glomerular filtration rate <60 ml/min per 1.73 m^2^, with lifestyle-related diseases such as hypertension, diabetes mellitus, and hyperlipidemia, with an acute illness in the preceding 6 weeks were excluded. Subjects routinely taking medications, such as birth control pills, were excluded. Among patients with CKD, those with cognitive decline or an inability to consent to participate in the study, psychiatric illness, oral lesions (tongue cancer and oral candida), active cancer (patients who had undergone within the past 5 years or were scheduled to receive systematic cancer treatment), active infections (antibacterial or antifungal infections requiring the systemic administration of antimicrobial agents), patients who are unable to cooperate with taste testing, patients with COVID19 or who were diagnosed with it after participation in the study, and other patients deemed inappropriate by the attending physician were excluded. The present study was conducted in accordance with the ethical guidelines of the Declaration of Helsinki and was approved by the Ethics Committee of Kyoto Prefectural University of Medicine (approval number ERB-C-2081). Written informed consent was obtained from all participants.

### Baseline Demographics and Clinical Characteristics

Baseline data on the following CKD patient confounders were obtained from medical records: age, sex, body mass index, comorbidities (diabetes, hypertension, dyslipidemia, and hyperuricemia), medications (renin-angiotensin system inhibitors, diuretics, and calcium channel blockers), and laboratory tests, which were performed within 2 weeks of the taste test. Creatinine, albumin, and electrolytes (sodium, potassium, chloride, phosphorus, calcium, zinc, and copper) were measured by a standard procedure. Estimated glomerular filtration rate was calculated according to the following formula^27^:

“194×(age[yr])−0.287×(serumcreatinine[mg/dl])−1.094×(0.739,iffemale).”

The coexistence of diabetes mellitus was defined as a history of glucose reduction treatment or a HbA1c value >6.5% by the National Glycohemoglobin Standardization Program. The coexistence of hypertension was defined as a history of antihypertensive treatment or an upper arm blood pressure >140/90 mm Hg. The coexistence of hyperlipidemia was defined as a history of antihyperlipidemic treatment or low-density lipoprotein cholesterol >140 mg/dl. Hyperuricemia was defined as a history of treatment or a uric acid level >7.0 mg/dl. Information on smoking, alcohol consumption, and denture use was collected using questionnaires completed by subjects at the time of taste testing. All baseline data on confounding factors for healthy subjects were obtained using questionnaires completed by subjects at the time of taste testing.

### Measurement of Gustatory and Aversion Thresholds

Assessments of gustatory and aversion thresholds were performed using a stimulant-impregnated test strip (Taste Disc, Sanwa Kagaku Kenkyusho Co., Ltd.), which is used in general clinical practice to evaluate taste disorders. Reagents were used at 5 concentrations as follows: sucrose (0.3%, 2.5%, 10%, 20%, and 80%) for the sweet taste, tartaric acid (0.02%, 0.2%, 2%, 4%, and 8%) for the sour taste, and quinine hydrochloride (0.001%, 0.02%, 0.1%, 0.5%, and 4%) for the bitter taste. Regarding the salt taste, we developed 9 levels of NaCl (0.3%, 0.6%, 0.9%, 1.2%, 1.5%, 2.0%, 5%, 10%, and 20%) to examine taste thresholds at finer concentrations. According to the previously described protocol of taste tests,^26^^,^^28^ participants placed filter paper with 1 drop of each of these reagents on their tongue in the order of the lowest to highest stimulant content. They were asked whether they tasted anything; if they replied affirmatively, they were asked the taste type and whether they disliked the taste or not. We asked the “dislike or not” question because, in contrast to taste preference tests using foods or beverages,^17^^,^^29^^,^^30^ we predicted that few people would “like” flavored filter paper; therefore, we decided to ask “dislike or not” instead of “like or not”. We defined the detection threshold for the lowest concentration sensed when participants tasted the reagents as the recognition threshold for the concentration that may be perceived as the taste type (salt, sweet, sour, and bitter tastes) and the aversion threshold as the concentration that was initially unpleasant. Furthermore, because filter paper itself may have a taste, we placed a piece of filter paper with no taste on the tongue as a control. Moreover, a strong or disliked taste that persists on the tongue may affect the accuracy of the evaluation; therefore, subjects rinsed their mouth with water before a change in the taste type.

### Statistical Analysis

Statistical analyses were performed using JMP Pro 16 (SAS Institute Inc., Cary, NC) to investigate whether relationships between variables were significant. Fisher exact test was used for nominal scales and the Wilcoxon test for continuous scales. A *P*-value <0.05 was considered to indicate a significant difference.

## Results

### Assessment of the Aversion Threshold for a High Salt Taste in Healthy Subjects

**Figure 1:**
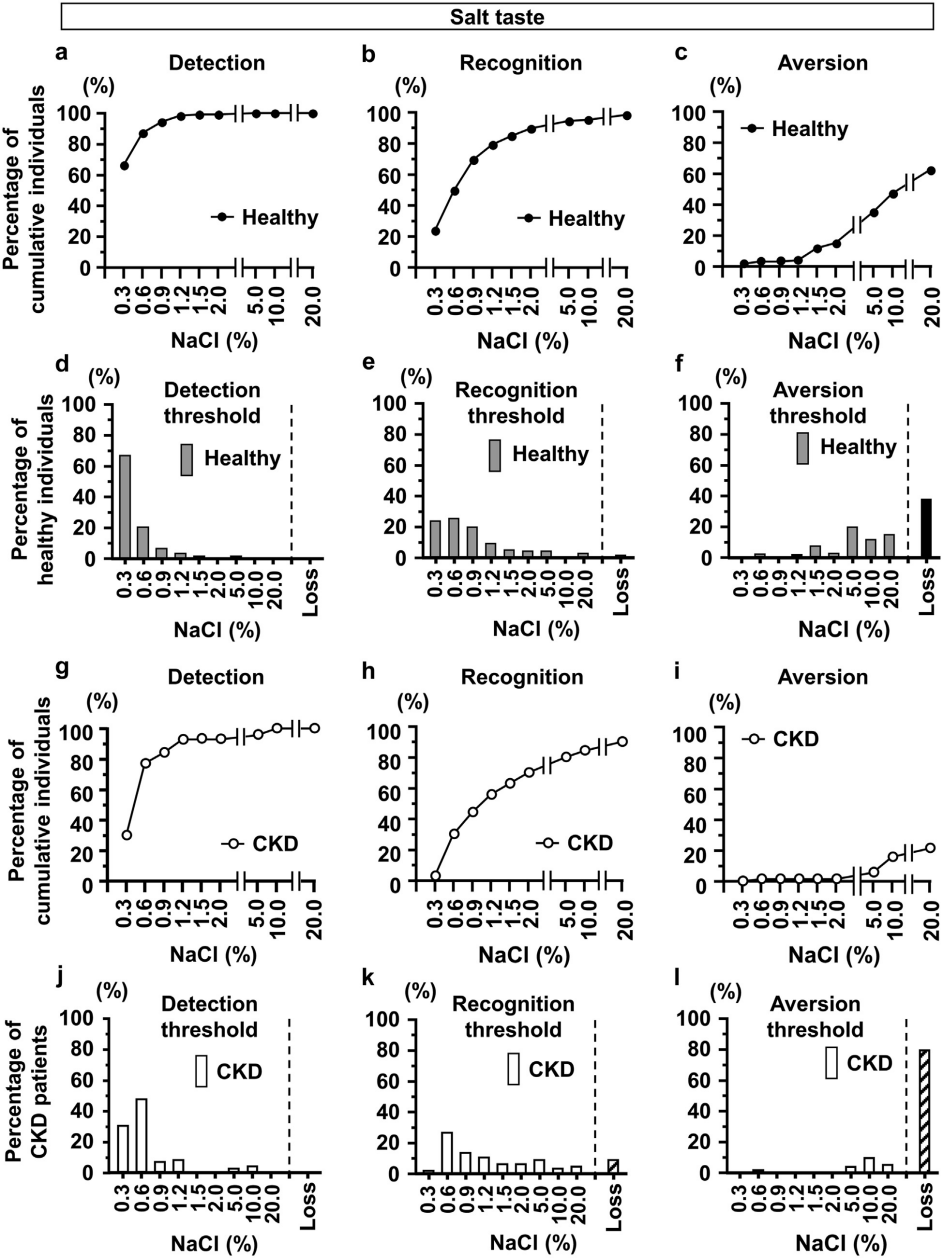
Detection, recognition. and aversion thresholds for NaCl in healthy volunteers and patients with CKD. Percentage of cumulative healthy subjects for the (a) detection of, (b) recognition of, and (c) aversion to various concentrations of NaCl. Percentage of healthy subjects with (d) detection, (e) recognition, and (f) aversion thresholds for various concentrations of NaCl. Percentage of cumulative patients with CKD for the (g) detection of, (h) recognition of, and (i) aversion to various concentrations of NaCl. Percentage of patients with CKD with (j) detection, (k) recognition, and (l) aversion thresholds for various concentrations of NaCl. Subjects unable to detect or recognize even the highest concentration of NaCl or who did not show avoidance to the highest concentration of NaCl were described as “Loss”. CKD, chronic kidney disease; NaCl, sodium chloride.

**Figure 2:**
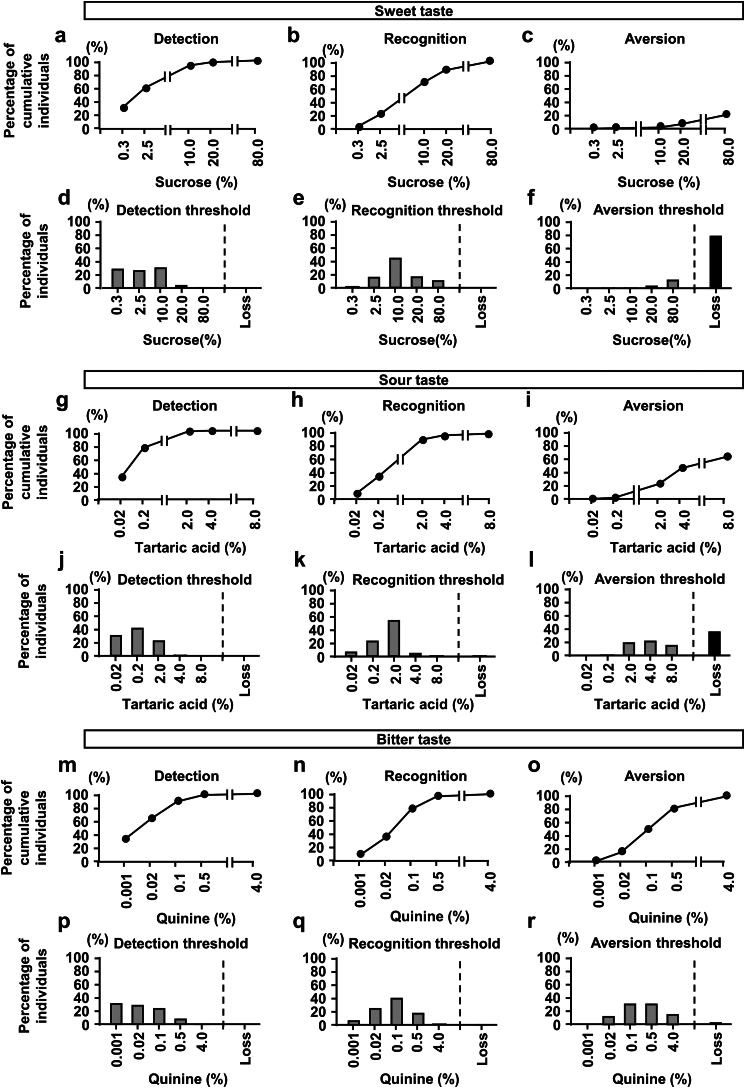
Detection, recognition, and aversion thresholds for sweet, sour, and bitter tastes in healthy volunteers. Percentage of cumulative individuals for the (a) detection, (b) recognition, and (c) aversion to various concentrations of sucrose. Percentage of individuals with (d) detection, (e) recognition, and (f) aversion thresholds for various concentration of sucrose. Percentage of cumulative individuals for the (g) detection, (h) recognition, and (i) aversion to various concentrations of tartaric acid. Percentage of individuals with (j) detection, (k) recognition, and (l) aversion thresholds for the various concentrations of tartaric acid. Percentage of cumulative individuals for the (m) detection, (n) recognition, and (o) aversion to various concentrations of quinine. Percentage of individuals with (p) detection, (q) recognition, and (r) aversion thresholds for various concentrations of quinine. Subjects unable to detect or recognize even the highest concentration of each taste or who did not show avoidance to the highest concentration of each taste were described as “Loss”.

As a pilot study, we performed taste tests on 125 healthy volunteers with a median age of 38 years and a high percentage of females (60%) (Table 1). We initially examined detection and recognition thresholds for the salt taste (Figure 1). Based on previous findings showing that the normal recognition threshold of a salt taste was 0.6%,^26^^,^^31^ 49.6% and 69.6% of healthy subjects recognized salt concentrations of 0.6% and 0.9%, respectively (Figure 1a, b, d, and e). Regarding the other tastes, more than 80% of subjects recognized up to 3 of the 5 concentrations (Figure 2a, b, d, e, g, h, j, k, m, n, p, and q).

**Table 1:** Characteristics of healthy subjects

Chracteristics	All (*N* = 125)	Aversion to 10% NaCl		*P-*value^a^
		yes (*n* = 59)	No (*n* = 66)	
Age, yr	38 (28–49)	33 (26–47)	40 (30.8–50.5)	0.0456
Sex (M/F)	50 / 75	22 / 37	28 / 38	0.5584
Body mass index (kg/m^2^)	21.2 (19.5–22.8)	21.0 (19.4–22.8)	21.2 (19.5–22.7)	0.7842
Smoking (Yes/No)	11 / 114	3 / 56	8 / 58	0.2138
Drinking (Yes/No)	68 / 57	33 / 26	35 / 31	0.1060
False teeth (Yes/No)	15 / 110	9 / 50	6 / 60	0.2898

F, female; NaCl, sodium chloride; m, male.

Continuous variables are presented as medians (interquartile ranges).

a Fisher exact test was used for nominal scales and the Wilcoxon test for continuous scales.

To examine aversion to the 4 taste senses, we performed taste tests with higher concentrations of each stimulant until subjects described the taste as unpleasant. Regarding the salt taste, aversiveness did not occur at lower concentrations, but became apparent from 1.5% NaCl and gradually increased in a concentration-dependent manner (Figure 1c and f). However, though the percentage of the loss of recognition of a salt concentration of 20% was only 1.6%, the loss of aversion, even to 20%, was noted in 37.6% of healthy subjects (Figure 1f). Concerning the sweet taste, few participants showed aversiveness, particularly at the highest concentration of sucrose (80%) (Figure 2c and f). In addition, when we asked the type of taste in every test strip, all participants answered that the test strip of the highest concentration of salt was “salty,” but not “bitter” or “sour.” Regarding the sour and bitter tastes, aversiveness also increased in a concentration-dependent manner, which was pronounced for the bitter taste (Figure 2i, l, o, and r). Previous studies did not sufficiently quantify the aversive response to a salt taste in human subjects. Therefore, based on the present results showing that the median NaCl concentration of aversiveness in healthy subjects was 10% (Figure 1c and f), we arbitrarily divided subjects according to this threshold and found no significant differences in their characteristics (Table 1).

### Disturbed Taste Senses and Aversion Thresholds for High Salt Concentrations in Patients With CKD

**Figure 3:**
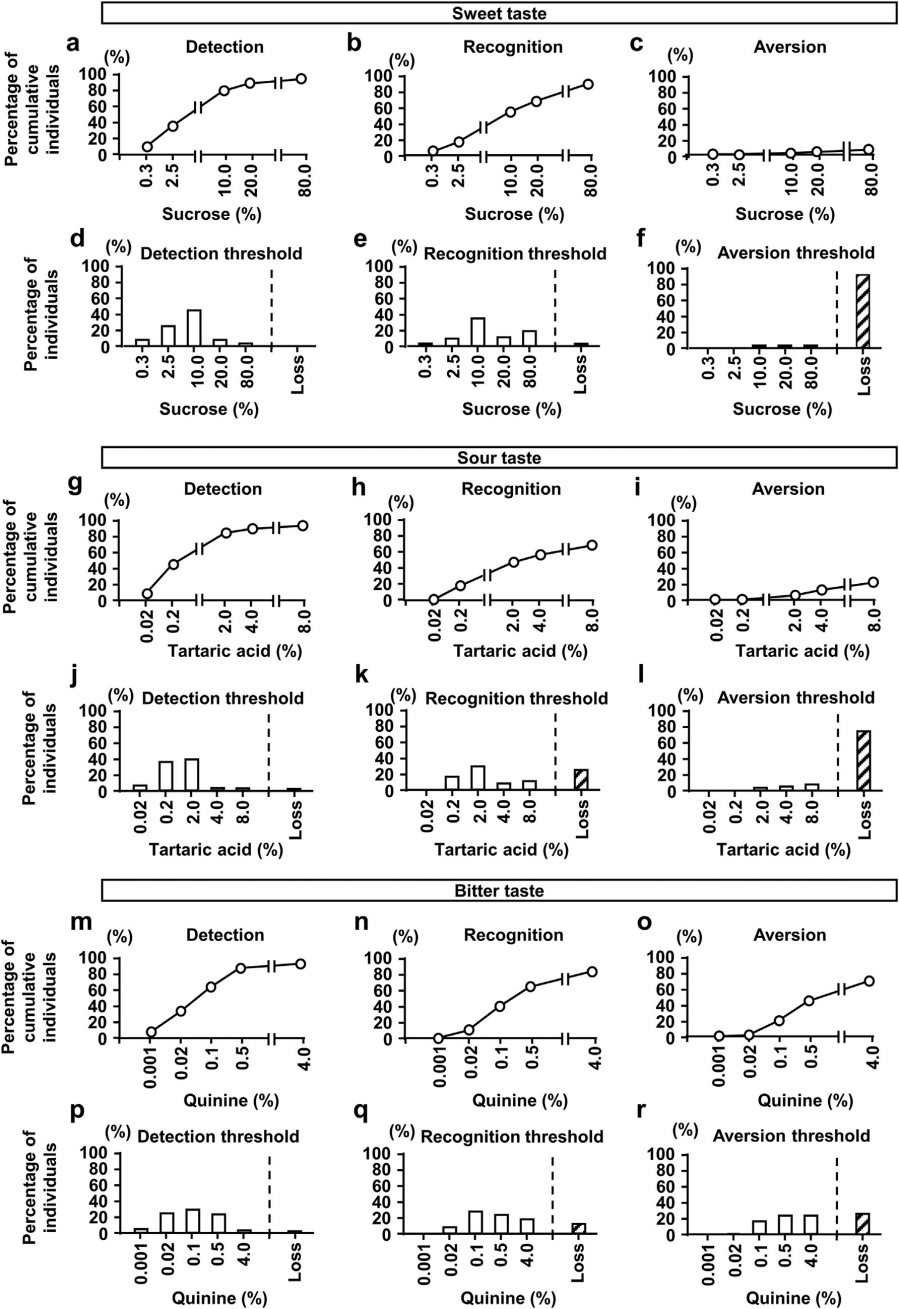
Detection, recognition, and aversion thresholds for sweet, sour, and bitter tastes in patients with CKD. Percentage of cumulative individuals for the (a) detection, (b) recognition, and (c) aversion to various concentrations of sucrose. Percentage of individuals with (d) detection, (e) recognition, and (f) aversion thresholds for various concentrations of sucrose. Percentage of cumulative individuals for the (g) detection, (h) recognition, and (i) aversion to various concentrations of tartaric acid. Percentage of individuals with (j) detection, (k) recognition, and (l) aversion thresholds for various concentrations of tartaric acid. Percentage of cumulative individuals for the (m) detection, (n) recognition, and (o) aversion to various concentrations of quinine. Percentage of individuals with (p) detection, (q) recognition, and (r) aversion thresholds for various concentrations of quinine. Subjects unable to detect or recognize even the highest concentration of each taste or who did not show avoidance to the highest concentration of each taste were described as “Loss”. CKD, chronic kidney disease.

We investigated whether taste senses and aversion thresholds were disturbed in patients with CKD. Patients with CKD were older than healthy subjects (median age of 66.5 years) and included a higher percentage of males (58.6%) (Table 2). Average creatinine was 2.22 mg/dl and 20.0% of participants had diabetes. Regarding the recognition threshold, only 29.9% and 44.2% of participants recognized salt concentrations of 0.6% and 0.9%, respectively (Figure 1g, h, j, and k), which was consistent with our previous findings.^26^ Furthermore, 10% of patients with CKD did not recognize the highest salt concentration of 20% NaCl (Figure 1h and k). Regarding other tastes, the detection and recognition of the sweet taste were similar to those of healthy subjects (Figure 3a, b, d, and e). The detection of the sour and bitter tastes was similar to that of healthy subjects (Figure 3g, j, m, and p), whereas their recognition was disturbed, with 27.1% and 14.1% of patients with CKD being unable to accurately recognize the sour and bitter tastes, respectively (Figure 3h, k, n, and q).

**Table 2:** Characteristics of patients with CKD

Characteristics	CKD (*N* =70)	yes (*N* = 11)	No (*N* = 59)	*P-*value^a^
Age, yr	66.5 (54.8–76)	73 (55–76)	66 (55–76)	0.5998
Sex (M/F)	41 / 29	2 / 9	39 / 20	0.0057
Body mass index (kg/m^2^)	23.3 (20.6–25.9)	20.8 (19.8–25.4)	23.8 (21.1–26.4)	0.0828
Smoking (Yes/No)	12 / 58	1 / 10	11 / 48	0.6752
Drinking (Yes/No)	35 / 35	3 / 8	32 / 27	0.1875
False teeth (Yes/No)	23 / 47	0 / 11	23 / 36	0.0122
Cr (mg/dl)	2.22 (1.22–5.60)	4.01 (0.77–5.66)	2.11 (1.28–4.02)	0.9165
eGFR (ml/min per 1.73 m^2^)	24.5 (9.83–47.3)	11.99 (8.76–75.4)	26.08 (12.2–43.8)	0.9936
Diabetes (Yes/No)	14 / 56	1 / 10	13 / 46	0.4424
Hypertension (Yes/No)	56 / 14	8 / 3	48 / 11	0.6814
Hyperlipidemia (Yes/No)	35 / 35	3 / 8	32 / 27	0.1875
Hyperuricemia (Yes/No)	35 / 35	5 / 6	30 / 29	1.00
Na (mmol/l)	139 (137–141)	141 (135–142)	139 (137–141)	0.9012
K (mmol/l)	4.2 (4.0–4.5)	4.3 (4.1–4.5)	4.2 (4.0–4.5)	0.6929
Cl (mmol/l)	104 (101–106)	105 (103–107)	104 (101–106)	0.5151
P (mg/dl)	3.7 (3.3–4.95)	3.75 (3.6–5.6)	3.65 (3.3–4.85)	0.1628
Ca (mg/dl)	8.9 (8.4–9.3)	8.7 (8.0–9.0)	8.9 (8.4–9.3)	0.1604
Alb (g/dl)	3.4 (3.0–3.9)	3.3 (2.7–3.5)	3.5 (3.0–3.9)	0.2211
Zn (μg/dl)	67.0 (59.8–81.3)	61 (46–82)	67 (60–81)	0.2231
Cu (μg/dl)	105.5 (83.8–122.3)	116 (86–134)	104 (83–118)	0.1496

Cr, creatinine; eGFR, estimated glomerular filtration rate; F, female; m, male.

Continuous variables are presented as medians (interquartile ranges).

a Fisher’s exact test was used for nominal scales and the Wilcoxon test for continuous scales.

Concerning aversiveness to the salt taste in patients with CKD, 78.6% of patients showed the complete loss of aversion to salt (Figure 1i and l). To examine the factors contributing to the reduced aversion to the salt taste, we divided patients with CKD into 2 groups based on the aforementioned median NaCl concentration of aversiveness in healthy subjects, and found that only 15.7% showed a normal aversion to NaCl. In terms of relevant patient characteristics, males and the use of dentures were identified as factors contributing to the reduced aversion to the salt taste (Table 2). Regarding the sweet taste, no aversion was observed in patients with CKD, which was similar to healthy subjects (Figure 3c and f). Aversion to the sour and bitter tastes was also reduced in patients with CKD, with 77.1% and 28.6% showing the complete loss of aversion to these tastes, respectively (Figure 3i, m, p, and s). Overall, the recognition of and aversion to the salt, sour, and bitter tastes were disturbed in patients with CKD.

## Discussion

The 5 fundamental taste senses: bitter, sweet, salt, sour, and umami, are generally preserved in animals as diverse as insects and mammals, and near-universally drive the consumption of nutrients and avoidance of toxins or harmful compounds.^11^ Mammals show a common taste preference, with an attraction for sweet and umami tastes and an aversion to bitter and sour tastes.^19^ In contrast to these tastes, animals have a unique response to the salt taste, namely, attraction to a low concentration and aversion to a high concentration. This biphasic response has mainly been investigated in rodents and insects,^10^ but remains unclear in humans. Therefore, the present study examined aversive responses to high concentrations of taste stimulants in healthy subjects and patients with CKD. Consistent with experiences in daily life, quantitative taste tests on healthy subjects showed aversive responses to high concentrations of salt, bitter, and sour stimulants. Taste tests on patients with CKD revealed that their recognition of the salt, sour, and bitter tastes were disturbed. Moreover, aversive responses to high salt, bitter, and sour concentrations were impaired in patients with CKD. Given the importance of an appropriate diet with a focus on salt reduction in the treatment of lifestyle-related diseases, these taste disorders in patients with CKD may affect dietary behavior, which makes appropriate dietary restrictions difficult to achieve.

Consistent with animal experiments, our quantitative analyses revealed aversive responses to high salt concentrations in humans. However, in contrast to animals, some individuals, even healthy subjects, exhibited the loss of aversiveness to high salt concentrations. In addition, though mouse experiments showed aversion to high salt concentrations via the activation of bitter and sour taste sensing cells,^15^ no participant answered that the highest salt concentration was “bitter” or “sour” even though they described the taste as unpleasant.

There are several explanations for the discrepancies observed in the findings of taste tests between humans and rodents. One explanation is differences in the examination protocol from animal taste tests. In rodent taste tests, the salt preference test is performed with preconditioning as a furosemide treatment and a salt-removed diet, whereas the salt aversive test is conducted under artificially free-water removed conditions.^12^^,^^15^^,^^19^^,^^32^ The former preconditioning is aimed at inducing the activation of renin-angiotensin system, which promotes salt attraction,^33^, ^34^, ^35^ whereas the latter is aimed at inducing high serum osmolarity, causing mice to avoid salt in order to prevent further increases in serum sodium concentrations.^35^ Both of these are considered to be instinctive behaviors in mice to maintain the homeostasis of body fluids and serum osmolarity, and are not generally applicable to taste preference or aversiveness during social activities in humans. Taste experiments with a similar pretreatment to rodent experiments have been conducted on humans; however, the findings obtained were complex. Beauchamp *et al.*^36^ reported that salt restriction and diuretics increased the preference for a salt taste in humans, similar to mice. On the other hand, Manevitz *et al.*^37^ demonstrated that the loss of NaCl from sweat due to exercise did not increase the preference for a salt taste. In addition, although preference experiments have been conducted,^24^ behavioral studies on aversiveness in human are quite limited.

Another explanation is the different motivations driving dietary behavior between humans and rodents, which may lead to inconsistencies in taste test results. This inconsistency may be attributed to human taste preferences being affected not only by instinctive “likes” and “dislikes,” as observed in mice, but also by acquired dietary behavior caused by region, culture, habits, and individual health conditions, including sickness, smoking habits, aging, and oral wellness. The effects of these factors on taste senses cannot be easily evaluated, and, thus, a large-scale human study will be required in the future.

Regarding taste tests in patients with CKD, we found that salt taste recognition as well as the aversive response to high salt were impaired in patients with CKD. Taste impairment is common in patients with CKD and its causes include aging, uremia, neuropathy in patients with diabetes mellitus, medications, a low serum zinc concentration, and impaired oral health.^17^^,^^23^^,^^26^ Uremic toxins in saliva have been suggested to damage taste cells in patients with CKD.^38^

In the present study, whereas 84.3% of all patients with CKD recognized 10% NaCl, only 15.7% exhibited an aversion to it, indicating a high percentage of patients who did not show aversiveness despite recognizing the high salt concentration. These differences between the recognition and aversive thresholds for NaCl suggest that an increase in the recognition threshold alone is not the only explanation for decreased aversiveness to the salt taste. The results obtained herein showed that being male and the usage of false teeth were factors related to aversiveness to high salt in patients with CKD. Sex differences have been noted in taste preferences, detection, and reactivity to taste stimuli in humans and rodents.^39^ The human threshold for stimulus detection appears to differ between sexes, with women detecting basic taste stimuli at lower concentrations than men.^39^ In addition, rat experiments showed that an aversive response to a high concentration of salt (1 M of NaCl) was more frequently observed in females than in males.^40^

Regarding the effects of oral health on taste sensations, possible oral sources of taste disorders include the following: dental caries, periodontal diseases, dental infections, tooth loss, poor oral hygiene, and dry mouth.^41^ Patients with CKD frequently develop oral disorders, and the salivary glands, periodontium, teeth, and oral mucosa may be affected, leading to oral manifestations, including early tooth loss and periodontitis.^42^ These disorders may affect the number of taste cells as well as the expression of various transmembrane taste channels on these cells, thereby affecting taste sensations in patients with CKD. A recent study on mice identified transmembrane channel 4^16^ as a candidate channel for the ENaC-independent pathway in taste cells. Although it remains unclear whether it is also expressed in humans and plays a role in the signaling pathway for a high salt taste, a similar channel is assumed to exist. If the expression of the channels responsible for salt taste aversion on taste cells is reduced in CKD, similar to other taste channels, the aversive response to a high salt taste may also be affected.

The present study has several limitations. Not only the concentration of, but also the total amount of stimulants clearly affects the intensity of taste. The total amount of stimulants impregnated in filter paper in the present study was smaller than in taste tests using foods or beverages, which may have resulted in an underestimation of taste sensations. Furthermore, the causal effect of the loss of aversiveness to a high salt taste on salt intake remains unknown. In addition, the backgrounds of patients with CKD differed from those of healthy subjects, and various confounders, such as age and sex, were included. Therefore, the contribution of renal dysfunction to the loss of an aversion to a high salt taste is unclear. Moreover, the present study was conducted at a single center in Japan and all participants were Asian. Taste may be strongly affected by dietary habits based on race, region, country, and social environment. Therefore, it is unclear whether the results obtained herein are a general phenomenon in humans regardless of their backgrounds.

In conclusion, the quantitative taste test revealed an aversive response to a high salt taste in humans. Approximately 40% of healthy subjects did not exhibit an aversive response to a salt concentration of 20%; patients with CKD showed even more impaired aversive reactions to high salt, sour, and bitter tastes. These results suggest that it is extremely difficult to rely on a patient’s own taste sense to adequately limit salt intake. It currently remains unclear whether the attenuation of salt taste aversion increases salt intake or if an enhancement in salt taste aversion by another method decreases salt intake. In addition, due to the small number of patients with CKD examined and the lower rate of normal aversion thresholds among them, the results obtained herein cannot fully explain the discrepancy between recognition and aversion thresholds and the relevant factors reducing salt aversion; therefore, future investigations are warranted.

## Disclosure

All the authors declared no competing interests.

## Supplementary Material

Supplementary File (PDF)

**STROBE checklist.**
