# Supplementary material for: Aversion to a High Salt Taste is Disturbed in Patients With CKD
Source: Kidney Int Rep. 2024 Feb 16;9(5):1254–64. doi: 10.1016/j.ekir.2024.02.1393 (PMC11069016; doi:10.1016/j.ekir.2024.02.1393)
Supplement: Supplementary File (PDF) [file mmc1.pdf]

STROBE Statement—checklist of items that should be included in reports of observational studies

	Item No.	Recommendation	Page No.	Relevant text from manuscript
**Title and abstract**	1	(*a*) Indicate the study's design with a commonly used term in the title or the abstract	N/A	
		(*b*) Provide in the abstract an informative and balanced summary of what was done and what was found	Manuscript, page 2	Assessments of gustatory and aversion thresholds for salt, bitter, sour, and sweet tastes were performed using a stimulant-impregnated test strip in healthy subjects and CKD patients. The present results confirmed the anticipated aversive response to a high salt taste in humans and demonstrated its impairment in CKD patients, implying that CKD patients have reduced resistance to a high salt intake.
**Introduction**				
Background/rationale	2	Explain the scientific background and rationale for the investigation being reported	Manuscript, page 3	Based on these findings, salt intake in mammals is assumed to be affected by the appetitive-aversive balance e.g. for low salt concentrations (accelerator) and the avoidance of high salt concentrations (decelerator). Most of the taste tests previously conducted on humans analyzed salt taste

				recognition or preferences under specific disease conditions 17, while not many quantitatively examined aversive responses to a high concentration of salt 18.
Objectives	3	State specific objectives, including any prespecified hypotheses	Manuscript, page 3-4	To elucidate the aversive behavior of salt, it is necessary to study not only the salt taste, but also its interactions with other tastes. In the present study, we attempted to quantitatively evaluate aversive behavior to high salt concentrations in healthy subjects by adding the concepts of the preference for and aversion to salt. Moreover, since taste disorders are common in CKD patients 17, 19-26, we performed taste tests on CKD patients.
**Methods**				
Study design	4	Present key elements of study design early in the paper	N/A	
Setting	5	Describe the setting, locations, and relevant dates, including periods of recruitment, exposure, follow-up, and data collection	Manuscript, page 5	Subjects section
Participants	6	(*a*) *Cohort study*—Give the eligibility criteria, and the sources and methods of selection of participants. Describe methods of follow-up<br>*Case-control study*—Give the eligibility criteria, and the sources and methods of case ascertainment and control selection. Give the rationale for the choice of cases and controls	Manuscript, page 5	Subjects section

		Cross-sectional study—Give the eligibility criteria, and the sources and methods of selection of participants		
		(*b*) *Cohort study*—For matched studies, give matching criteria and number of exposed and unexposed<br>*Case-control study*—For matched studies, give matching criteria and the number of controls per case	N/A	
Variables	7	Clearly define all outcomes, exposures, predictors, potential confounders, and effect modifiers. Give diagnostic criteria, if applicable	Manuscript, page 5-6	Baseline demographics and clinical characteristics section<br><br>Measurement of gustatory and aversion thresholds section
Data sources/ measurement	8*	For each variable of interest, give sources of data and details of methods of assessment (measurement). Describe comparability of assessment methods if there is more than one group	Manuscript, page 5-6	Baseline demographics and clinical characteristics section<br><br>Measurement of gustatory and aversion thresholds section
Bias	9	Describe any efforts to address potential sources of bias	N/A	
Study size	10	Explain how the study size was arrived at	N/A	

Continued on next page

Quantitative variables	11	Explain how quantitative variables were handled in the analyses. If applicable, describe which groupings were chosen and why	N/A	
Statistical methods	12	(*a*) Describe all statistical methods, including those used to control for confounding	Manuscript, page 7	Statistical analysis
		(*b*) Describe any methods used to examine subgroups and interactions	N/A	
		(*c*) Explain how missing data were addressed	N/A	
		(*d*) *Cohort study*—If applicable, explain how loss to follow-up was addressed<br>*Case-control study*—If applicable, explain how matching of cases and controls was addressed<br>*Cross-sectional study*—If applicable, describe analytical methods taking account of sampling strategy	N/A	
		(*e*) Describe any sensitivity analyses	N/A	
**Results**				
Participants	13*	(a) Report numbers of individuals at each stage of study—eg numbers potentially eligible, examined for eligibility, confirmed eligible, included in the study, completing follow-up, and analysed	Manuscript, page 8	For healthy volunteers, described in Table 1.<br>For CKD patients, described in Table 2
		(b) Give reasons for non-participation at each stage	N/A	
		(c) Consider use of a flow diagram	N/A	
Descriptive data	14*	(a) Give characteristics of study participants (eg demographic, clinical, social) and information on exposures and potential confounders	Manuscript, page 8	As indicated in Table 1 and Table 2
		(b) Indicate number of participants with missing data for each variable of interest	N/A	
		(c) *Cohort study*—Summarise follow-up time (eg, average and total amount)	N/A	
Outcome data	15*	*Cohort study*—Report numbers of outcome events or summary measures over time	Figures 1 to 3	As indicated in Figures 1 to 3
		Case-control study—Report numbers in each exposure category, or summary measures of exposure	N/A	
		Cross-sectional study—Report numbers of outcome events or summary measures	N/A	
Main results	16	(*a*) Give unadjusted estimates and, if applicable, confounder-adjusted estimates and their precision (eg, 95% confidence interval). Make clear which confounders were adjusted for and why they were included	Figures 1 to 3 Tables 1 and 2	As indicated in Figures 1 to 3 and Tables 1 and 2
		(*b*) Report category boundaries when continuous variables were categorized	N/A	

	(*c*) If relevant, consider translating estimates of relative risk into absolute risk for a meaningful time period	N/A

Continued on next page

Other analyses	17	Report other analyses done—eg analyses of subgroups and interactions, and sensitivity analyses	N/A	
**Discussion**				
Key results	18	Summarise key results with reference to study objectives	Manuscript, page 10	Consistent with experiences in daily life, quantitative taste tests on healthy subjects showed aversive responses to high concentrations of salt, bitter, and sour stimulants. Taste tests on CKD patients revealed that their recognition of the salt, sour, and bitter tastes were disturbed. Moreover, aversive responses to high salt, bitter, and sour concentrations were impaired in CKD patients.
Limitations	19	Discuss limitations of the study, taking into account sources of potential bias or imprecision. Discuss both direction and magnitude of any potential bias	Manuscript, page 12	The present study has several limitations. Not only the concentration of, but also the total amount of stimulants clearly affects the intensity of taste. The total amount of stimulants impregnated in filter paper in the present study was smaller than in taste tests using foods or beverages, which may have resulted in an underestimation of taste sensations. Furthermore, the causal effect of the loss of aversiveness to a high salt taste on salt intake remains unknown. In addition, the backgrounds of CKD patients differed from those of healthy subjects, and various

				confounders, such as age and sex, were included. Therefore, the contribution of renal dysfunction to the loss of an aversion to a high salt taste is unclear. Moreover, the present study was conducted at a single center in Japan and all participants were Asian. Taste may be strongly affected by dietary habits based on race, region, country, and social environment. Therefore, it is unclear whether the results obtained herein are a general phenomenon in humans regardless of their backgrounds.
Interpretation	20	Give a cautious overall interpretation of results considering objectives, limitations, multiplicity of analyses, results from similar studies, and other relevant evidence	Manuscript, page 12	In conclusion, the quantitative taste test revealed an aversive response to a high salt taste in humans. Approximately 40% of healthy subjects did not exhibit an aversive response to a salt concentration of 20%; CKD patients showed even more impaired aversive reactions to high salt, sour, and bitter tastes. These results suggest that it is extremely difficult to rely on a patient's own taste sense to adequately limit salt intake. It currently remains unclear whether the attenuation of salt taste aversion increases salt intake or if an enhancement in salt taste aversion

				by another method decreases salt intake. In addition, due to the small number of CKD patients examined and the lower rate of normal aversion thresholds among them, the results obtained herein cannot fully explain the discrepancy between recognition and aversion thresholds and the relevant factors reducing salt aversion; therefore, future investigations are warranted.
Generalisability	21	Discuss the generalisability (external validity) of the study results	N/A	
**Other information**				
Funding	22	Give the source of funding and the role of the funders for the present study and, if applicable, for the original study on which the present article is based	Manuscript, page 14	Competing interests and sources of funding

*Give information separately for cases and controls in case-control studies and, if applicable, for exposed and unexposed groups in cohort and cross-sectional studies.
